# Revealing the active microbiome connected with the rhizosphere soil of maize plants in Ventersdorp, South Africa

**DOI:** 10.3897/BDJ.9.e60245

**Published:** 2021-02-25

**Authors:** Olubukola O. Babalola, Rebaona R. Molefe, Adenike E. Amoo

**Affiliations:** 1 Food Security and Safety Niche, Faculty of Natural and Agricultural Sciences, North-West University, Private Bag X2046, Mafikeng, South Africa Food Security and Safety Niche, Faculty of Natural and Agricultural Sciences, North-West University, Private Bag X2046 Mafikeng South Africa

**Keywords:** Illumina sequencing, metagenomics, rhizosphere, maize plants

## Abstract

We conducted shotgun metagenomics sequencing of the maize rhizosphere and bulk soils in Ventersdorp, South Africa. Information on the structural composition and functional capabilities of microbial communities in the maize rhizosphere are provided by the data. Characterising the functional potentials of rhizosphere microbiomes gives an opportunity to link the microbiome to plant growth and health and provides the possibility of discovering new plant-beneficial genes that could enhance agricultural sustainability.

## Introduction

Maize is one of South Africa's most economically-valuable crops. Globally, it fills the diets of billions of people with basic carbohydrates. Poor management practices, such as over-fertilisation, have gone up significantly due to the quest to feed the ever-increasing human population. Therefore, it is imperative to identify eco-friendly fertilisers that do not have adverse effects on soil and maize development. Plants establish associations with soil microorganisms for various functions including nutrient cycling, stress tolerance and pathogen immunity (Liu et al. 2019). Increased knowledge of these mechanisms is a productive and positive way for the improvement of sustainable agriculture (Babalola et al. 2021).

The rhizosphere, which is the medium between plants and soil, has been labelled a 'hotspot' for new genes and biomolecules (Babalola et al. 2020). Plant-root exudates generate nourishing conditions for microbial growth and easily attract a selection of soil microorganisms (Adedeji and Babalola 2020; Canarini et al. 2019; Chukwuneme et al. 2021). Microbial communities in the rhizosphere are recruited from the large and diverse pool of microbes in bulk soils through root exudate chemical signalling (Adedeji and Babalola 2020;Hartman and Tringe 2019). This has contributed to an increase in microbial activity and quantity in rhizosphere soils compared to bulk soils. In contrast, microbial diversity significantly reduces in the rhizosphere soil relative to bulk soil (Praeg et al. 2019; Hartman and Tringe 2019).

Rhizosphere microbes exist to protect against pathogens and improve growth by developing phytohormones. These organisms enable plants to handle environmental disruptions, such as irregular climate-related changes in temperature, drought and salinity (Lu et al. 2018). It has been shown that nitrogen-fixing rhizobia and the mycorrhizal fungi in the rhizosphere have significant impacts on plant nutrient status (Mendes et al. 2013; Lu et al. 2018). For example, symbiotics, such as mycorrhizal fungi, are important for the absorption of nutrients and minerals from the soil to plants. Therefore, studies on the rhizospheric microbes and their functions could open several appealing features, from alleviating several of the consequences of climate change and environmental stress on plants by modifying plant features using microbial inocula to enhancing crop production. Therefore, the discovery of new genes in the maize rhizosphere could be an incentive to fix food insecurity and promote agricultural sustainability.

### Value of the dataset

The dataset contains raw sequences (FASTQ format files) obtained using shotgun metagenomic sequencing of the maize rhizosphere and bulk soils. Samples were collected from the maize rhizosphere (F3R1) and bulk (F3B1) soils to understand the microbial community structure, function and plant-beneficial genes in maize plantations. These data can be used alone or along with other datasets to achieve a larger scale view with more power for maize-associated microbiome research.

## Methods

### Sampling

Soil samples were collected from the rhizosphere soil (F3R1) and the bulk soil (F3B1) of maize plants on 16 June 2019 from a farm situated at Ventersdorp, South Africa. The rhizosphere soil samples were collected at 8 cm diameter, 15 cm depth of maize plants. The bulk soils were also collected within the maize farms.

#### Environmental profile

The maize field being investigated in this study is a private farm in Ventersdorp in the North West Province of South Africa. The farm was intentionally selected, based on the geographic location and the availability of maize plants. Ventersdorp has summer temperatures ranging from 17ºC to 31ºC and winter temperatures ranging from 3ºC to 21ºC. The annual rainfall ranges between 300 mm and 600 mm with more rain falling in summer than in winter.

#### Geographic range

Ventersdorp, North West Province (approximately 26°19'36.9''S, 26°53'19.1''E). Coordinates: -26°18'60.00''S; 26°48'59.99''E.

### Sample processing

The soil samples were transported to the laboratory on ice and stored until further use. Genomic DNA extraction was conducted using the DNeasy PowerSoil^®^ DNA isolation kit (MoBio Laboratories, Carlsbad, CA) in accordance with the manufacturer's directions. The extracted DNA was sent for shotgun metagenome sequencing to the Molecular Research Laboratory (www.mrdnalab.com) in Texas, USA. The initial concentration of DNA was evaluated using the Qubit^®^ dsDNA HS Assay Kit (Life Technologies). The libraries were prepared using Nextera DNA Flex library preparation kit (Illumina), following the manufacturer's user guide. Using 50 ng of DNA from each sample, libraries were prepared according to the Illumina NovaSeq DNA library preparation protocol. The determination of library average insert size was determined using the Agilent 2100 Bioanalyzer (Agilent Technologies). The library insert size ranged from 617 bp to 873 bp. The libraries were pooled, diluted (to 0.6 nM) and sequenced paired-end for 300 cycles using the NovaSeq system (Illumina).

### Data processing

The raw metagenome sequences were subjected to quality control using Metagenomic Rapid Annotations using Subsystems Technology (MG-RAST) online server (Meyer et al. 2008). This resulted in evacuation of artificial sequences generated by sequencing errors, exclusion of sequences of host-specific organisms, unclear base filtering (abolition of sequences of > 5 questionable base pairs with a cut-off score of 15 Q) and filtering of length (abolition of sequences of > 2 standard deviations from mean length). Following the quality control (QC), the sequences were annotated using the BLAT (the BLAST-like alignment tool) algorithm (Kent 2002) against the M5NR database (Wilke et al. 2012), which provides a non-redundant integration of many databases. For taxonomic profiling of microbial communities, the SEED subsystem was used and evaluation of their functional profiles was performed using SEED subsystem level 1. The subsystem database revealed bacteria (98.76%) had the highest taxonomical representation compared with eukaryote (0.72%) and archaea (0.73%). Annotation revealed that F3R1 had 15,713,893 sequences totalling 2,338,704,495 bp size and 64.11% G+C content. F3B1 had 12,463,113 sequences totalling 1,850,061,852 bp size and G+C 66.11%.

#### Technologies used

MG-RAST (https://mg-rast.org).

Source: The National Human Genome Research Institute (NHGRI)

## Biodiversity scope

The maize rhizosphere soil sample had more microorganisms than the bulk soil sample.

### Target

The rhizosphere microbiome and their functional potentials.

### Taxonomic range

All soil microbiomes were identified to genus or species level. The study revealed that the most abundant phyla were Proteobacteria and Actinobacteria in the rhizosphere and bulk soils. Ascomycota and Basidiomycota were distributed fungal reads, while Thauarcheota and Euyarchaeota were distributed as archaeal reads, respectively, but with an abundance of < 1%.Table 1

### Functional range

The functional annotation using SEED subsystems revealed that reads were more ascribed to carbohydrates metabolism (15.76 to 15.90%), amino acids and derivatives (11.53 to 11.61%) and clustering-based systems (13.63 to 13.78%) in the maize rhizosphere and bulk soils samples.

## Data Resources

Maize associated microbiome studies (Suppl. material 1).

## Usage Rights

Creative Commons Public Domain Waiver (CC-Zero)

Usage Rights

## Supplementary Material

6F29AB80-87B9-51CA-8EB3-E2400FEAC47B 10.3897/BDJ.9.e60245.suppl1 Supplementary material 1 BioSample metadata file Data type Metagenomic data File: oo_464224.csv https://binary.pensoft.net/file/464224 Olubukola O. Babalola; Rebaona R. Molefe; Adenike E. Amoo

## Figures and Tables

**Table 1.** Taxonomic classification of microorganisms in the maize rhizosphere and bulk soils

**Domain**	**Phyla**	**F3R1**	**F3B1**
Bacteria	Acidobacteria	368735	226709
Bacteria	Actinobacteria	2511153	2794515
Bacteria	Aquificae	15800	10882
Bacteria	Bacteroidetes	347509	261611
Bacteria	Candidatus Poribacteria	3480	1944
Bacteria	Chlamydiae	6644	4512
Bacteria	Chlorobi	35848	24915
Bacteria	Chloroflexi	203916	170908
Bacteria	Chrysiogenetes	2318	1452
Bacteria	Cyanobacteria	190459	138395
Bacteria	Deferribacteres	6452	4206
Bacteria	Deinococcus-Thermus	66466	56965
Bacteria	Dictyoglomi	4479	3216
Bacteria	Elusimicrobia	1759	1224
Bacteria	Fibrobacteres	1452	1021
Bacteria	Firmicutes	393062	304682
Bacteria	Fusobacteria	5707	4073
Bacteria	Gemmatimonadetes	153957	140020
Bacteria	Lentisphaerae	4635	3029
Bacteria	Nitrospirae	27120	15636
Bacteria	Planctomycetes	185528	121559
Bacteria	Proteobacteria	3443718	2362316
Bacteria	Spirochaetes	17790	12214
Bacteria	Synergistetes	9010	6373
Bacteria	Tenericutes	1687	1121
Bacteria	Thermotogae	15919	11866
Bacteria	Verrucomicrobia	179156	100394
Bacteria	unclassified (derived from Bacteria)	22169	18869
Fungi	Ascomycota	30221	31523
Fungi	Basidiomycota	3615	2657
Fungi	Blastocladiomycota	14	20
Fungi	Chytridiomycota	44	32
Fungi	Glomeromycota	38	6
Fungi	Microsporidia	121	57
Fungi	unclassified (derived from Fungi)	43	20
Archaea	Crenarchaeota	10423	7705
Archaea	Euryarchaeota	59001	45925
Archaea	Korarchaeota	728	451
Archaea	Nanoarchaeota	60	44
Archaea	Thaumarchaeota	6596	5229
Viruses	unclassified (derived from Viruses)	1244	1109

## Hosting institution

North-West University
